# Acute Skin Toxicities After Ultrahypofractionated Versus Hypofractionated Postoperative Radiotherapy in Patients With Early Breast Cancer—A Single‐Institution Clinical Study

**DOI:** 10.1155/ijbc/3641929

**Published:** 2026-05-30

**Authors:** Gordana Petkovska, Ivica Ratosa, Valentina Bojovska Trajanovska, Marina Iljovska, Emilija Lazareva, Antonija Lazarevska, Violeta Klisarovska, Snezhana Smichkoska

**Affiliations:** ^1^ University Clinic for Radiotherapy and Oncology Skopje, Skopje, North Macedonia; ^2^ Faculty of Medicine, University of Ss. Cyril and Methodius, Skopje, North Macedonia, ukim.edu.mk; ^3^ Division of Radiotherapy, Institute of Oncology Ljubljana, Ljubljana, Slovenia, onko-i.si; ^4^ Faculty of Medicine, University of Ljubljana, Ljubljana, Slovenia, uni-lj.si

**Keywords:** breast cancer, radiodermatitis, radiotherapy, ultrahypofractionation

## Abstract

**Background:**

Real‐world safety data from Southeastern Europe are limited for ultrahypofractionated (UHF) whole‐breast irradiation (WBI). We aimed to evaluate acute skin toxicity with UHF versus hypofractionated (HF) postoperative radiation therapy in early breast cancer, following implementation of this fractionation regimen.

**Methods:**

This combined prospective–retrospective study included women aged ≥50 years following breast‐conserving surgery (pT1–2, pN0–1, M0). The UHF cohort (prospective) received 26 Gy in 5 fractions over one week between 2023 and 2024. The control group consisted of retrospective data obtained from institutional records of patients who received WBI using a HF schedule of 40.5–42.6 Gy in 15–16 fractions over 3 weeks between 2015 and 2020. All patients were treated with 3D‐CRT WBI without regional nodal irradiation or boost. Acute skin toxicity was graded by CTCAE v5.0 at end of treatment and at 4 and 12 weeks.

**Results:**

This study included 80 patients, with 40 in each group, with a mean age of 61.1 ± 6.6 years. Baseline clinicopathological characteristics were comparable, except for a slightly larger median tumor size in the UHF group (*p* = 0.037). At the end of radiation therapy, acute radiodermatitis occurred in 36 of 40 (90%) patients receiving UHF and in 38 of 40 (95%) patients receiving HF (p = 0.396). The grade distribution was comparable across the two groups (*p* = 0.53), and no grade ≥3 events were recorded. At 4 weeks, no adverse skin reactions were observed in patients receiving UHF, and by twelve weeks, no active radiodermatitis persisted in either group.

**Conclusions:**

UHF WBI (26 Gy/5 fractions) demonstrated comparable acute skin toxicity to standard HF regimens, supporting its safety and clinical feasibility in routine practice.

## 1. Introduction

Radiotherapy represents a fundamental component in the multimodality management of malignant diseases. It is estimated that over 50% of patients diagnosed with cancer receive radiotherapy at some point during their treatment course [1]. In breast cancer, radiotherapy is commonly employed in the adjuvant (postoperative) setting, but it may also serve as a definitive or palliative treatment modality.

Radiotherapy‐related adverse effects are broadly categorized as acute or late. Acute reactions occur within days of treatment initiation, typically affecting rapidly proliferating tissues such as the bone marrow, skin, and mucosa. Late effects develop months after therapy completion and involve slowly renewing tissues. Distinguishing between these two categories is important, as it influences the understanding and management of radiotherapy‐induced morbidity [2]. Acute skin toxicity commonly manifests as radiation dermatitis, occurring in up to 90% of patients, with more than 30% experiencing moderate to severe reactions [3]. Radiation dermatitis may significantly impair quality of life and, in severe cases, necessitate dose reduction or interruption of therapy. The associated emotional distress should also be acknowledged as a relevant aspect of patient care [4].

The occurrence of acute radiodermatitis is influenced by several factors. Patient‐related factors include body mass index (BMI) > 25kg/m^2^, the volume of the organ being irradiated (e.g. volume of the breast > 1300 cm^2^), localization of the volume being irradiated (e.g., skin fold in the axilla), smoking and the use of a tumor bed boost or bolus during treatment [5, 6]. Diabetes Mellitus and other metabolic comorbidities are also an established risk factor for impaired epidermal repair [7]. Factors related to the radiotherapy treatment itself include the total irradiation dose, the type (electrons vs. photons) and quality of the radiation beam (kilovoltage vs. megavoltage), as well as the size of the tumor volume being irradiated [8].

Fractionation is a recognized determinant of radiodermatitis incidence. Radiobiologically, acute skin reactions are less sensitive to fraction size than late‐responding tissues; consequently, lower total doses are anticipated to lessen both the severity and duration of these reactions. In the first trials designed to compare conventional fractionation (CF) and hypofractionation (HF), START A and START B [9–11], acute toxicity was not the primary endpoint, and detailed acute skin data are limited. However, summaries and reviews of the program, as well as other studies evaluating similar fractionation regimens [12, 13], have consistently reported no increase—and in some cases a reduction—in acute skin toxicity with HF schedules.

FAST Forward clinical trial, which examined UHF in the period 2011–2014, opened a new chapter in the treatment of early‐stage breast cancer and raised the importance of ultra‐HF [14]. In this study, two acute skin toxicity substudies were undertaken to test the safety of the tested schedules with respect to early skin reactions. In the first substudy, Grade 3 (G3) or higher RTOG toxicity rates were 13.6% for 40 Gy/15 fractions, 9.8% for 27 Gy/5 fractions, and 5.8% for 26 Gy/5 fractions. In the second substudy, G3 or higher CTCAE toxicity rates were 0%, 2.4%, and 0% for the same respective schedules. G2 toxicities in the second group were reported at 51%, 27%, and 36%, respectively [15]. The results from 10‐year follow‐up were presented on the European Society for Radiotherapy and Oncology (ESTRO) Congress 2025, showing ipsilateral breast tumor recurrence (IBTR) of 3.6% for the control group 40 Gy, 3.0% for the 27‐Gy group, and 2.0% for 26‐Gy group [16], confirming the previous 5 years results [14]. When UHF schedule was compared with HF schedule, hazard ratio was 0.62, indicating noninferiority and a trend toward superiority. Clinician and patient‐reported late normal tissue effects confirmed previous data that 26 Gy is comparable with 40 Gy, but the 27 Gy schedule has a higher rate of firmness, shrinkage, and induration on the irradiated breast [16].

The COVID‐19 pandemic unexpectedly accelerated global experience with the 5‐fraction UHF, delivering up to 26 Gy [17]. To facilitate implementation during a period of restricted hospital access and reduced treatment capacity, the FAST‐Forward trial investigators made all study materials—including treatment planning, dosimetry, and protocol documentation—freely available online [18]. Subsequently, this approach received formal endorsement in major clinical guidelines [19–21].

The experience with shorter fractionation regimens worldwide and global evidence base for acute skin toxicities with UHF radiotherapy in breast cancer is widely expanding (Table 1).

**Table 1:** Clinical trials analyzing acute skin toxicities in patients with early breast cancer treated with UHF schedule.

Study	*n*	Grade 0 (%)		Grade 1 (%)		Grade 2 (%)		Grade 3 (%)		Scale	Boost
		UHF	HF	UHF	HF	UHF	HF	UHF	HF		
Petkovska et al. (current study) [22]	80	10	5	82.5	80	7.5	15	0	0	CTCAE 5.0	No
FAST Forward^a^ [14]	352	6/6	0/0	62/58	32/49	27/36	55/51	6/0	14/0	RTOG/CTCAE 4.03	11%–17%/no
YO‐HAI5^b^ [23]	400			40.7–44	54.5–57.4	13–16.2	20.2–23.2	0		CTCAE 4.03	90.50%
Ratosa et al. [24]	276	41.8		50.4		7.8		0		CTCAE 5.0	58.30%
HYPORT^c^ [25]	271					2.96	11	1.1		CTCAE 5.0	38.70%
Dzhugashvili et al. [26]	242	56.1		43.1		0.8		0			60.70%
Calvo Tudela et al. [27]	160	21.3		73.1		5		0.6		CTCAE 4.0	16.30%
Laughlin et al. [28]	107					3.7	7.4	0	0	HCS	20.80%
Magdy et al. [29]	92	84.6	30	15.4	64	0	6	0	0	RTOG	Yes
Ivanov et al. [30]	60	45.5	61.5	63	45.5	18.5	3	0	0	CTCAE 5.0	No
Sigaudi et al. [31]	59	79.9		18.6		1.7		0		CTCAE 5.0	No

Abbreviations: CTCAE, common terminology criteria for adverse events; HCS, Harvard cosmesis scale; HF, hypofractionated group; *n*, number; RTOG, toxicity criteria of the radiation therapy oncology group; UHF, ultrahypofractionated group.

^a^FAST forward [14].

^b^YO‐HAI5 [23].

^c^HYPORT [25].

The study was initiated in 2023 alongside the introduction of UHF for patients with early breast cancer at the University Clinic for Radiotherapy and Oncology, Skopje, North Macedonia. It was designed as a real‐world replicating study from a setting where UHF was newly introduced. The study is aimed at assessing the safety of 3D conformal radiotherapy (3D‐CRT) using two different fractionation schedules, with a focus on the incidence of acute skin toxicity.

## 2. Methods and Materials

This study employs a combined prospective and retrospective design. Before starting the research, interventional protocol was created and approved by the Institutional Review Board and Ethical Committee at the Faculty of Medicine, Skopje. The study was conducted at the University Clinic for Radiotherapy and Oncology, Skopje, Republic of North Macedonia.

### 2.1. Patients

The inclusion criteria for the whole group of patients were the following: women, older than 50 years, after breast‐conserving surgery with defined axillary node status, invasive or in situ breast cancer, complete resection of the tumor (R0), tumor size up to 5 cm without chest wall or skin infiltration (pT1–2), and 0–3 positive lymph nodes (pN0–1). Concurrent hormone therapy or targeted anti‐HER2 therapy was permitted. Patients with ipsilateral invasive breast cancer, breast reconstruction surgery with implants, concurrent chemotherapy, residual microscopic disease (R1), tumors bigger than 5 cm (T3), tumors infiltrating the chest wall or the skin or inflammatory breast cancer (pT4), patients with four or more positive lymph nodes (pN ≥ 2), patients with other diagnosed and treated malignant disease in the last 5 years, and patients with contralateral invasive or in situ breast cancer in the last 5 years or metastatic disease were excluded from the study.

In the intervention group (prospective arm), patients were treated with WBI with UHF, to a total dose of 26 Gy in 5 fractions delivered in one week in the period of 2023 and 2024. The control arm consisted of retrospective data obtained from institutional records of patients who received WBI using a HF schedule of 40.5 Gy in 15 fractions (after 2019) or 42.6 Gy in 16 fractions (2015–2019) over 3 weeks. Notably, 20 patients from the HF group (50%) had previously participated in а clinical trial at the University Clinic for Radiotherapy and Oncology, Skopje, corresponding to the 42.6 Gy/16 fraction regimen.

### 2.2. Treatment Planning, Delivery, and Follow‐Up

Planning computed tomography (CT) was the same for the patients in both groups. CT scan was done in free breathing, in the supine position with both arms over the head, on an inclined simulation table with a breast board. All patients were treated with whole breast‐only radiation therapy, without regional nodes irradiation, and without tumor bed boost. Target volumes and organs‐at‐risk (OARs) were delineated according to the Radiation Therapy Oncology Group (RTOG) atlas [30] and 3D‐CRT plans were carried out according to institutional protocols with Varian Eclipse (Varian, Palo Alto, California) software. Portal verification was done every day in the UHF group and once a week in the HF group.

Planning target volume for evaluation (PTVeval) was described as a structure 5 mm under the chest skin, subtracted from PTV. Target volumes were optimized as follows: PTVeval_95*%* > 95*%* (the volume of PTVeval receiving 95% of the prescribed dose as a percentage), PTVeval_max*%* ≤ 110*%*, PTVeval_105*%* ≤ 5 cm^3^, and PTVeval_107*%* ≤ 2 cm^3^ (the volumes receiving 105% and 107% of the prescribed dose are expressed as percentages, respectively). Dose constraints for OARs included an ipsilateral lung volume receiving 8 Gy (LungV8 [*%*]) < 15*%*for UHF and 18 Gy (LungV18 [*%*]) < 25*%* for HF. For the heart volume receiving 1.5 Gy (HeartV1.5 [*%*]) < 30*%* and 20 Gy (HeartV20 [*%*]) < 10*%*, were applied for the UHF and HF group, respectively.

Acute skin toxicity was evaluated at the end of treatment and at 4‐ and 12‐week intervals posttreatment by the lead investigator (G.P.) for patients in the interventional group, using grading from the Common Terminology Criteria for Adverse Events (CTCAE) (Version 5.0) (Table 2). Data from institutional records of patients randomized to the control (HF) group were used to assess the severity of acute dermatitis at the end of treatment and 12 weeks after completion of therapy, in accordance with the institutional follow‐up protocol.

**Table 2:** The Common Terminology Criteria for Skin Adverse Events (CTCAE) Version 5.0.

Grade	Skin toxicity
0	No changes
I	Faint erythema or dry desquamation
II	Moderate or sharp erythema, uneven moist desquamation, mostly confined to skin folds and folds, moderate edema
III	Confluent moist desquamation in areas other than skin folds and folds, bleeding cause by minor trauma or abrasion
IV	Ulceration of the dermis, spontaneous bleeding, full thickness skin necrosis
V	Death

Additionally, dosimetric evaluation on the mean skin dose was performed. Data were obtained from the dose–volume histograms for patients in both groups. Equivalent dose in 2‐Gy fractions (EQD2) was calculated for all treatment plans using the formula *E* *Q* *D*2 = *D* *x*((*d* + (*α*/*β*)/(2 + *α*/*β*))) (D = total dose, d = dose per fraction, *α*/*β* = alpha/betaratio for tissue sensitivity [Gy]), assuming *α*/*β* ratio of 10 Gy for acute skin toxicities.

### 2.3. Statistical Analysis

The statistical analysis was done in the statistical program Statistical Package for the Social Sciences (SPSS Inc, Chicago, Illinois) Version 25.0. Average, minimum, and maximum values, standard deviation, median, and interquartile range are used to display quantitative qualities, whereas absolute and relative numbers are used to display qualitative features. Bivariate analysis was performed to compare the two radiation techniques. For comparison of the two techniques in terms of qualitative features, the Pearson chi‐square test and Fisher exact test were used, whereas for the analysis and comparison of quantitative features, the Student’s *t*‐test and Mann–Whitney test were used. Values of *p* < 0.05 were considered statistically significant.

## 3. Results

This clinical study included 80 patients between 50 and 75 years old (61.1 ± 6.6 years) according to the inclusion and exclusion criteria listed above. Baseline characteristics of the patient and tumor are listed in Table 3.

**Table 3:** Patient and tumor characteristics.

Parameter	*N*(%)	UHF	HF
**Age (years)** median (range)	(61.1 ± 6.6) (50–75)	60.8 ± 7.1	61.35 ± 6.2
**Breast cancer laterality**			
Right	38 (47.5)	22 (55)	16 (40)
Left	42 (52.5)	18 (45)	24 (60)
BMI (mean ± SD) (min–max)	(27.2 ± 3.6) (20.2–35)	27.9 ± 3.7	26.4 ± 3.3
**Diabetes mellitus**			
Yes	7 (8.75)	4 (12.9)	3 (9.1)
No	57 (71.25)	27 (87.1)	30 (90.9)
Missing information	16 (20)		
**Histopathology**			
NST	56 (70)	29 (72.5)	27 (67.5)
Lobular	11 (13.75)	6 (15)	5 (12.5)
Mixed	4 (5)	2 (5)	2 (5)
Others	7 (8.75)	1 (2.5)	6 (15)
Тis	2 (2.5)	2 (5)	0
**PT**			
1b	7 (8.75)	2 (5.26)	5 (12.5)
1c	37 (46.25)	18 (47.37)	19 (47.5)
1mi	3 (3.75)	0	3 (7.5)
2	31 (38.75)	18 (47.37)	13 (32.5)
**Size (mm)** (mean ± SD) (min–max)	(18.11 ± 8.2) (1–45)	20.3 ± 8.2 (7–45)	16.3 ± 7.9 (1–35)
**PN+**	5 (6.25)	2 (5.26)	3 (7.5)
**Stage**			
I	47 (58.75)	20 (52.63)	27 (67.5)
II	31 (38.75)	18 (47.37)	13 (32.5)
Other	2 (2.5)		
**G**			
1	10 (12.5)	3 (8.11)	7 (17.5)
2	51 (63.75)	27 (72.97)	24 (60)
3	16 (20)	7 (18.92)	9 (22.5)
Missing information	3 (3.75)		
**LV**			
Present	42 (52.5)	21 (61.76)	21 (56.76)
Absent	29 (36.25)	13 (38.24)	16 (43.24)
Missing information	9 (11.25)		
**IHC**			
ER+ Her+	8 (10)	4 (10.53)	4 (10)
ER+ Her−	59 (73.75)	31 (81.58)	28 (70)
ER− Her+	3 (3.75)	0	3 (7.5)
ER− Her−	8 (10)	3 (7.89)	5 (12.5)
**PR**			
<1%	18 (22.5)	7 (20.59)	11 (27.5)
1%–10%	6 (7.5)	5 (14.71)	1 (2.5)
>10%	50 (62.5)	22 (64.71)	28 (70)
Missing information	6 (7.5)		
**Chemotherapy**	28 (35)	14 (36.84)	14 (35)
**Hormonal therapy**	70 (87.5)	36 (94.74)	34 (85)
**Targeted therapy**	13 (16.25)	6 (15.79)	7 (17.5)
	14		

Abbreviations: BMI, body mass index; ER, estrogen receptor; G, grade; HER2, human epidermal growth factor receptor 2; IHC, immunohistochemical type; LV, lymphovascular invasion; NST, nonspecific type; pN+, positive lymph nodes; PR, progesterone receptor; pT, tumor stage; tis, tumor in situ; UHF, HF.

The distribution between the patient’s and tumor’s characteristics was even between the two groups. No statistical significance was found according to patients’ age, tumor side, BMI, presence of diabetes mellitus, adjuvant treatment with chemotherapy, hormonal therapy, and targeted therapy, breast size, *T* stage, *N* stage, lymphovascular invasion, grade, histological, or immunohistochemical type. Only a statistical difference was noted according to tumor size, where the UHF‐RT group showed a median difference of 4 mm bigger than the HF‐RT group (*p* = 0.037).

The mean BMI was slightly higher in the UHF group compared with the HF group (27.9 ± 3.7 vs. 26.4 ± 3.3), with median values of 27.5 (IQR 25–28.4) and 26.1 (IQR 24.1–28.4), respectively; however, this difference did not reach statistical significance (*p* = 0.13).

A total of 7 patients (10.9%) had diabetes, including 4 (12.9%) in the UHF group and 3 (9.1%) in the HF group. The distribution of diabetes was similar between groups, with no statistically significant difference (*χ* ^2^ = 0.24, *p* = 0.625).

According to breast size patients were divided in two groups: small volume (<1300 cm^3^) and large volume (>1300 cm^3^), which is shown in Table 4. The majority of patients in both groups had a smaller treated volume: 36 (90%) patients in the investigation group (UHF) and 32 (80%) patients in the control group (HF).

**Table 4:** Clinical target volume size.

Clinical target volume size	Group			*p*level
	*n*	UHF *n* (%)	HF *n* (%)	
<1300 cm^3^	68	36 (90)	32 (80)	*X* ^2^ = 1.57
>1300 cm^3^	12	4 (10)	8 (20)	*p* = 0.21
All	80	40	40	

Abbreviations: HF, hypofractionated group; *n*, number UHF, ultrahypofractionated group.

**Figure 1:**
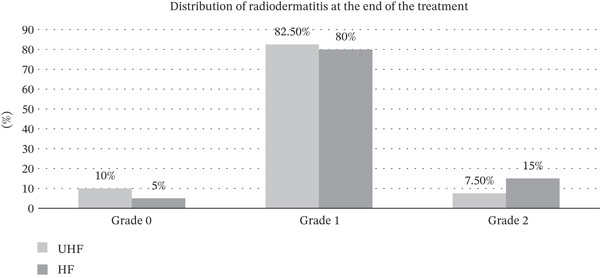
Distribution of radiodermatitis at the end of treatment. Abbreviations: UHF, ultra‐hypofractionated group; HF, hypofractionated group.

Acute skin reactions (radiodermatitis) at the end of treatment were observed in 36 patients (90%) in the UHF group and 38 patients (95%) in the HF group. The distribution of patients with and without radiodermatitis did not differ significantly between the groups, although a slightly higher incidence was noted in the HF group (*p* = 0.396). Similarly, no statistically significant difference was found between the groups regarding radiodermatitis grade (G) (*p* = 0.53). The distribution of radiodermatitis grades is presented in Figure 1. No severe ≥ G3 skin reactions or serious adverse events related to skin toxicity were observed in either group.

At the 4‐week follow‐up, no patients in the UHF group exhibited delayed acute radiodermatitis, and by week 12, no radiodermatitis was observed in either group.

The UHF group received significantly lower mean skin doses (27.86 vs. 36.58 Gy), with a statistically significant difference (*p* < 0.001) (Table 5).

**Table 5:** Dosimetric evaluation on the mean skin dose.

Group	*D* *m* *e* *a* *n* *s* *k* *i* *n* *E* *Q* *D*2*G* *y*_*α*/*ß* = 10				*p*level
	*n*	*M* *e* *a* *n* ± *S* *D*	Min–max	Median (IQR)	
UHF	40	27.9 ± 0.5	26.6–29.9	28 (27.7–28.2)	
HF	40	36.6 ± 0.8	34.9–38.4	36.6 (36.02–36.9)	*p* < 0.001^∗^

Abbreviations: D mean skin EQD2Gy, mean skin dose expressed in equivalent dose to 2 Gy; HF, hypofractionated group; *n*, number; UHF, ultrahypofractionated group.

## 4. Discussion

In the current real‐world study, we assessed the acute toxicity of a UHF schedule compared with HF during its implementation in postoperative irradiation for selected patients with early breast cancer in North Macedonia. Our findings revealed no statistically significant difference in the rate of acute cutaneous reactions at the end and at the 3‐month follow‐up after radiation therapy.

These findings are consistent with previously published data from many studies which have demonstrated comparable or reduced rates of acute skin toxicity with HF and UHF regimens. Table 1 presents a brief summary of the most relevant publications that explored UHF. From a radiobiological perspective, this observation is supported by the lower total dose delivered in UHF schedules, which may reduce the severity and duration of acute skin reactions despite the larger dose per fraction.

Compared with other studies, our findings show a higher incidence of G1 radiodermatitis (82.5%). The rates of G2 reactions and the absence of G3 radiodermatitis are consistent with those reported in most published studies. The severity of radiation dermatitis is commonly evaluated using standardized grading systems such as RTOG [32] and CTCAE [33]. However, despite their standardized criteria, grading may vary due to the subjective interpretation of terms like “faint erythema” or “moderate desquamation.” In our study, data from both groups indicate a tendency to assign G1 radiodermatitis more frequently than G0.

An international multidisciplinary consensus endorsed by ESTRO outlined essential requirements for reporting radiotherapy in breast cancer clinical trials [34]. According to these recommendations, the use of the CTCAE v5.0 grading system is advised. However, as shown in Table 1, different trials have used varying grading criteria to report skin toxicity. Even within the FAST‐Forward substudy, RTOG criteria were applied to the initial cohort, whereas subsequent patients were assessed using CTCAE criteria [15]. Such inconsistency complicates direct comparison between studies and reduces the reliability of cross‐trial evaluations.

An important limitation of the present study is the asymmetry in follow‐up between the treatment groups. The UHF cohort was evaluated prospectively at the end of treatment, as well as at 4 and 12 weeks, whereas the HF control group was assessed retrospectively only at the end of treatment and at 12 weeks. As a result, observations at the 4‐week time point in the UHF group do not have a corresponding comparator in the HF cohort, precluding any direct comparison at this interval.

Nevertheless, the inclusion of a 4‐week assessment in the UHF cohort allows for the observation of delayed acute radiodermatitis, a clinically relevant phenomenon increasingly described in the literature, typically manifesting 4–8 weeks after completion of a 5‐fraction regimen. Real‐world data from the Institut Curie [35] have highlighted this pattern in patients treated with the FAST‐Forward protocol and suggest the importance of routine clinical evaluation at approximately 1‐month posttreatment. In the present study, no cases of delayed radiodermatitis were observed in the UHF group at 4 weeks; however, this finding should be interpreted with caution in light of the lack of corresponding data in the HF group, as well as the limited sample size.

BMI and diabetes mellitus represent clinically relevant modifiers of radiation‐induced skin toxicity [6]. Although these variables were balanced between groups in the present study and did not demonstrate a statistically significant impact, their potential influence should be acknowledged when interpreting toxicity profiles.

The dosimetric analysis of mean skin dose demonstrated a highly statistically significant difference between the groups, as expected. These findings are consistent with previous studies [22, 30] evaluating the dosimetric characteristics of UHF radiotherapy in breast cancer patients and likely reflect the lower total dose and the use of more conformal treatment techniques associated with this regimen.

Beyond clinical outcomes, the implementation of UHF has important implications for resource utilization and healthcare delivery, particularly in radiotherapy centers with limited capacity, such as the University Clinic for Radiotherapy and Oncology in Skopje, which serves as the only reimbursed radiotherapy center in North Macedonia. A reduction in the number of treatment fractions directly decreases the use of radiotherapy equipment, staff workload, and overall operational demands. In addition, indirect benefits for patients should be considered, including fewer hospital visits, reduced transportation costs, less time away from work, and improved overall comfort [36].

The principal strengths of this study include its pioneering implementation of UHF for breast cancer in North Macedonia, contributing valuable regional real‐world data where evidence has been limited. The results from this real‐world replication study are contributing to the growing body of evidence supporting its clinical adoption, particularly in underrepresented geographic regions. In the study, the majority of the patients were followed prospectively, and it ensures consistency with the reporting of the adverse events in the prospective group since one investigator followed up all patients. Several limitations should be acknowledged, including the relatively small sample size and certain methodological limitations: the single‐institution setting, differences in follow‐up assessment between the groups, and the absence of baseline skin assessment prior to radiotherapy initiation.

In conclusion, the results of this study corroborate the acute skin toxicity data of ultrashort radiation therapy for patients undergoing whole‐breast radiation therapy following surgery for early‐stage breast cancer. The similar occurrence of acute radiodermatitis in both participant groups (HF vs. UHF) suggests that a shorter regimen of whole breast radiotherapy is a feasible treatment option and should be considered a standard of care for selected patients.

## Author Contributions

G.P. contributed to the conception and design of the study, patient recruitment, data collection, clinical evaluation, analysis and interpretation of results, and drafting of the manuscript. I.R. contributed to the critical revision of the manuscript. V.B.T., M.I., E.L., A.L., and V.K. contributed to patient management, data collection, radiotherapy treatment planning and delivery, interpretation of clinical findings, and revision of the manuscript for important intellectual content. S.S. contributed to the conceptual development of the study, supervision, statistical interpretation, and manuscript preparation. All authors reviewed and approved the final version of the manuscript and agreed to be accountable for all aspects of the work.

## Funding

No funding was received for this manuscript.

## Disclosure

The authors have nothing to report.

## Ethics Statement

This study was conducted in accordance with the ethical standards of the institutional and national research committees and with the 1964 Declaration of Helsinki and its later amendments. The protocol was reviewed and approved by the Institutional Review Board and the Ethics Committee of the medical faculty, Skopje, North Macedonia. Written informed consent was obtained from all participants in the prospective cohort prior to inclusion in the study.

## Conflicts of Interest

The authors declare no conflicts of interest.

## Data Availability

The data that support the findings of this study are available on request from the corresponding author. The data are not publicly available due to privacy or ethical restrictions.
